# Chest Pain and Palpitations After Spinal Surgery: An Atypical Presentation of Acute Cholecystitis

**DOI:** 10.1155/cris/5037012

**Published:** 2026-05-09

**Authors:** Jordan Purewal, Jason Silvers, Jared Gennett, Michael Knight, Nasim Ahmed, Xiu Sun

**Affiliations:** ^1^ Department of Surgery, Jersey Shore University Medical Center, Neptune, New Jersey, USA, jerseyshoreuniversitymedicalcenter.com; ^2^ St. Georges University School of Medicine, West Indies, St. Georges, Grenada, sgu.edu; ^3^ Department of Pathology, Jersey Shore University Medical Center, Neptune, New Jersey, USA, jerseyshoreuniversitymedicalcenter.com

## Abstract

Acute cholecystitis is a common disease that affects ~200,000 people in the United States each year. Early diagnosis and gallbladder removal are associated with improved patient outcomes, including fewer postoperative complications, shorter length of stay, and lower hospital costs. Acute cholecystitis typically presents with right upper quadrant (RUQ) pain associated with nausea and vomiting. The pathophysiological mechanism of RUQ pain corresponds with dermatomes that arise from thoracic spinal cord levels T6 to T8. We describe a case of a 40‐year‐old male who presented with a 2‐day history of substernal chest pain, which was worked up for acute coronary syndrome and was later found to have acute cholecystitis. This atypical presentation was attributed to the patient’s history of a paraspinal schwannoma after resection with laminectomy, complicated by persistent right hemi‐thoracoabdominal paresthesia. We aim to shed light on this atypical presentation of acute cholecystitis.

## 1. Introduction

Acute cholecystitis typically presents with right upper quadrant (RUQ) pain. In contrast to biliary colic, where the pain subsides after a few hours, patients with acute cholecystitis have persistent pain. Associated with the pain, patients can have nausea, vomiting, fevers, and anorexia [1]. We present a case of a male who was initially evaluated for acute coronary syndrome and was subsequently found to have acute cholecystitis. Unique to this patient was his history of a schwannoma resection in the past, which resulted in persistent numbness of the RUQ and presentation of acute cholecystitis with substernal pain.

## 2. Case Report

The patient was a 40‐year‐old gentleman with a past medical history of class 1 obesity. His past social history included a 25‐pack‐year smoking history. His past surgical history was significant for a T7 laminectomy with excision of a right paraspinal schwannoma in 2018. He presented to the emergency department with a history of 2 days of substernal chest pain, intermittent palpitations, and chest heaviness. He described the substernal chest pain as persistent, 5/10 (moderate on the numeric rating scale) in intensity, and radiating to the right back. The patient stated he had self‐resolving episodes of chest pain in the past and could not recall any associated factors or patterns of his symptoms. He was never formally worked up for these prior episodes, so it is unknown if these were cardiac in origin or prior episodes of gallbladder attacks. He denied fevers, chills, nausea, vomiting, lightheadedness, or dizziness. The patient noted that since he had his spinal surgery in 2018, he has had impaired sensation over the right side of the right anterior chest and abdomen.

Upon initial evaluation, the patient was tachycardic with a heart rate of 120 beats/min and a blood pressure of 152/96 mmHg. His body temperature was 98.8°F with a respiratory rate of 18 breaths/min. The patient was in no acute distress. His neck was supple with no jugular venous distention. He had decreased breath sounds in the lower lung fields but no rales on auscultation. S1 and S2 heart sounds were present on cardiac examination without any murmurs or gallops. The patient’s abdomen was soft, nondistended, and nontender. There was no sensation in the skin on the right abdomen. The hallmark finding of inspiratory pause on deep palpation of the RUQ from pain, Murphy’s sign, was not elicited in the patient’s physical exam.

**Figure 1:**
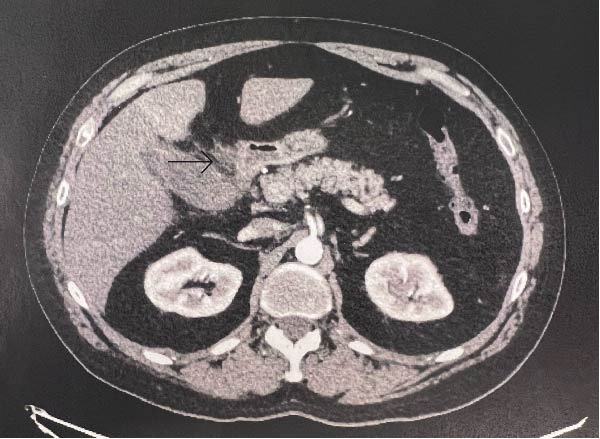
CT scan showing pericholecystic fat stranding (arrow).

A complete blood count showed a white blood cell count of 9.8 K/uL. The electrocardiogram showed normal sinus rhythm without ST‐T wave changes, and the troponin I value was <0.01 ng/mL (normal 0–0.04 ng/mL). Total bilirubin was 0.8 mg/dL (normal 0.2–1.3 mg/dL), alkaline phosphatase was 97 U/L (normal 38–126 U/L), AST was 38 U/L (normal 10–42 U/L), and ALT was slightly elevated at 64 U/L (normal 10–60 U/L). A computed tomography angiogram (CTA) of the chest, abdomen, and pelvis was performed, as there was concern for a thoracoabdominal aortic dissection on his initial workup. The scan showed no aortic disease; however, the gallbladder was noted to be mildly dilated with pericholecystic fat stranding and enhancement of the gallbladder wall (Figure 1).

**Figure 2:**
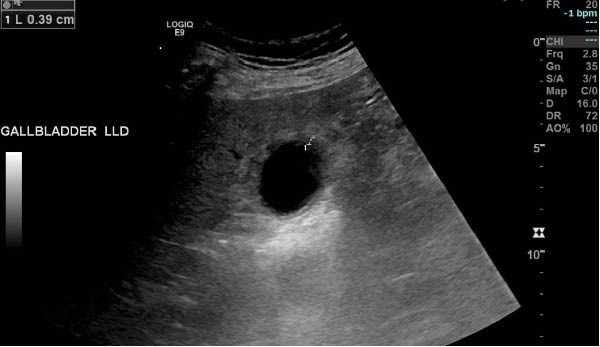
Ultrasound showing gallbladder wall thickness (0.39 cm).

**Figure 3:**
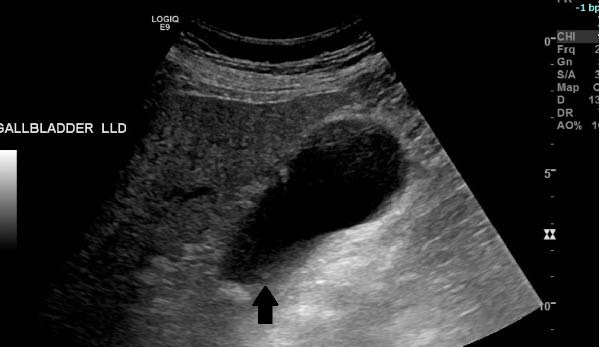
Right upper quadrant ultrasonography showing a distended gallbladder with cholelithiasis (arrow).

A follow‐up RUQ ultrasound also showed a dilated gallbladder with 4 mm wall thickness (normally less than 3 mm) and cholelithiasis (Figures 2 and 3).

**Figure 4:**
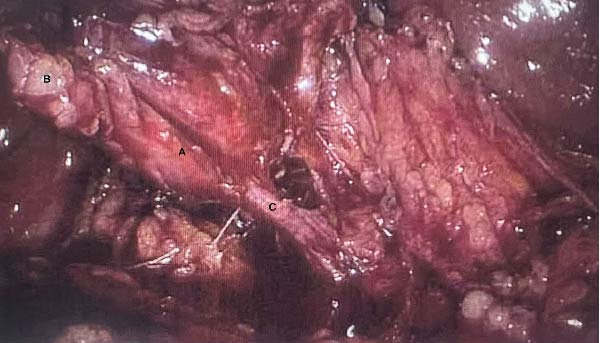
Intraoperative image of the critical view of safety. A Inflamed gallbladder, B omental fat adherent to gallbladder, and C cystic duct.

The patient was taken to the operating room on the day of presentation for a laparoscopic cholecystectomy. The patient was found to have an inflamed gallbladder with omentum encasing the gallbladder (Figure 4).

**Figure 5:**
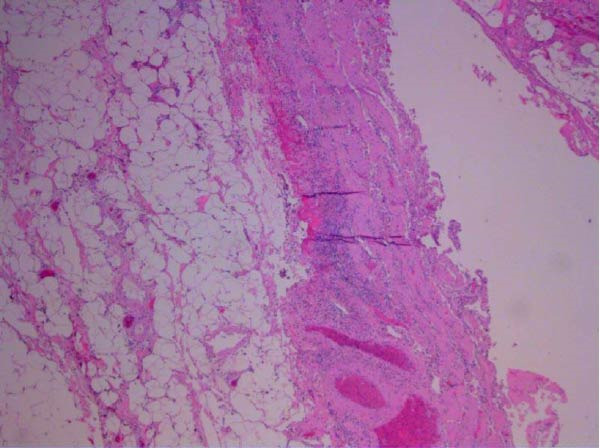
Gallbladder wall with marked surface erosion, mural acute inflammation, congestion, and hemorrhage (5×).

**Figure 6:**
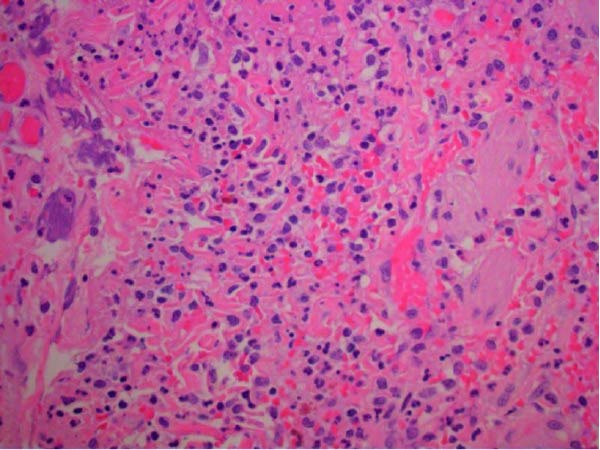
Many neutrophils and a few lymphocytes in the muscularis propria of the gallbladder (40×).

The gallbladder was removed without complication. The specimen was sent to pathology for examination and found to have an ovoid‐shaped green/tan gallbladder stone impacted in the gallbladder neck measuring 3.0 cm × 2.5 cm × 1.7 cm. No polyp, mass, or other lesion was identified. The gallbladder wall thickness ranged from 1to 4 mm. The tissue examination was consistent with acute cholecystitis (Figures 5 and 6).

After an uneventful postoperative course, the patient was discharged home the next morning without chest pain. Consent for publishing this case report and allowing for imaging studies to be used was obtained by telephone from the patient.

## 3. Discussion

The classic presentation of acute cholecystitis consists of RUQ pain and tenderness, frequently nausea and vomiting, and a positive Murphy’s sign, which is an inspiratory arrest with palpation of the RUQ [1]. Acute cholecystitis may also present in atypical patterns such as chest pain and EKG changes. Obesity and pregnancy can also have an impact on symptoms and physical examination [2]. Presentation of acalculous acute cholecystitis secondary to ischemia of the gallbladder wall usually occurs in severely ill patients with fever and right hypochondrium tenderness [3]. Excessive visceral stimulation from biliary disease, triggering a vagal reflex (cardio–biliary reflex), was likely involved in the patient’s tachycardia on initial presentation. Although the imaging findings in this case were consistent with gallbladder pathology, the complete absence of both local and systemic signs of inflammation meant the case failed to meet the threshold of even the “suspected” diagnosis of cholecystitis under the Tokyo Guidelines 2018 (TG18) diagnostic criteria for acute cholecystitis [4].

Pain in the RUQ from gallbladder disease arises because of referred pain from the viscera to the somatic location of the RUQ. Pain from gallbladder disease can also commonly be referred to the right shoulder and subscapular region. The anterolateral/spinothalamic sensory pathway carries the sensation of pain and temperature from the peripheral nervous system, synapses with second‐order central neurons, which then cross the midline and carry pain information to the brain. The phenomenon of referred pain is due to nerves that innervate visceral organs arising from the same spinal cord segments that innervate along a dermatomal distribution [5]. The T7 dermatome distribution is in the bilateral upper quadrants of the abdomen. The patient described above, who had a T7 paraspinal intercostal nerve schwannoma resection with laminectomy, may have presented without RUQ pain or tenderness secondary to these somatosensory pathways being disrupted.

Spinal cord injuries have been linked to an increased incidence of cholecystitis [6]. Tandon et al. [7], Moonka et al [8], and Tola et al. [9] describe an increase in biliary sludge and cholelithiasis. Rotter and Larrain [10] hypothesize that this increase in gallstones in spinal cord injury patients may be a result of colonic dysmotility secondary to the injury of the spinal cord, which in turn causes a deoxycholic acidemia and an increase in gallstone formation. Kim et al. [11] investigated the neurologic impairment following schwannoma resection. They found that the majority of patients did not have deficits postoperatively corresponding to the dermatome of the nerve root resected. In this study, the schwannoma was removed from either levels C5–T1 or L3–S1 [11].

Maeda et al. [12] outlined three primary neural pathways through which biliary disease can cause chest pain mimicking cardiac disease. These pathways include nociceptive signals traveling via the sympathetic afferent fibers of mainly T4–T6 from the gallbladder and bile ducts, vagal reflexes following excessive visceral stimulation, and inflammatory signals via the phrenic nerve [12].

Our patient’s schwannoma was removed from the T7 level, with a lack of sensation at that dermatome level, which may be the reason that he did not present with typical RUQ pain. Because of the atypical presentation, our patient underwent an extensive workup, including a CTA of the chest, abdomen, and pelvis to exclude the differential diagnosis of aortic dissection. The CTA showed findings that alerted the ED provider to further investigate acute cholecystitis. In patients presenting with chest pain, there is a risk of framing bias, which is the clinician being influenced by the initial context or chief complaint. There can also be anchoring bias, where there is a fixation on ruling out cardiac disease, which can delay the diagnosis of other pathologies, including abdominal pathologies. Efforts should be taken by clinicians to avoid these diagnostic pitfalls. Strategies such as a differential diagnosis checklist and intentional pauses to reflect (diagnostic time‐outs) can be effective.

Elective laminectomy, particularly one with the removal of a schwannoma, and subsequent loss of dermatome sensation can bring challenges to the diagnosis of common diseases, such as in this case of acute cholecystitis. Point‐of‐care physicians have to be aware of these rare conditions and atypical presentations of common diseases.

## Author Contributions

Jordan Purewal, Jason Silvers, and Jared Gennett collaborated to write this manuscript and review the relevant literature. Michael Knight was the primary surgeon involved in the case and, along with Nasim Ahmed, revised and prepared the manuscript. Xiu Sun provided the pathology for the manuscript.

## Funding

No funding was received for this manuscript.

## Ethics Statement

This study was carried out in accordance with the Code of Ethics of the World Medical Association (Declaration of Helsinki). As per HMH IRB, case reports do not need IRB approval.

## Consent

Written informed consent was obtained from the patient for the publication of this case report and accompanying images. A copy of the written consent is available for review upon request.

## Conflicts of Interest

The authors declare no conflicts of interest.

## Data Availability

Data sharing does not apply to this study, as no new data were created or analyzed in this study.
